# COVID-19 pneumonia in lung transplant recipients: understanding risk factors and treatment outcomes in Japan

**DOI:** 10.1007/s10238-024-01388-y

**Published:** 2024-06-10

**Authors:** Toshikazu Watanabe, Takashi Hirama, Miki Akiba, Tatsuaki Watanabe, Yui Watanabe, Hisashi Oishi, Hiromichi Niikawa, Yoshinori Okada

**Affiliations:** 1 Department of Respiratory Medicine, Fujita Health University School of Medicine, Toyoake, Aichi Japan; 2 Department of Thoracic Surgery, Institute of Development, Aging and Cancer, Tohoku University, Sendai, Miyagi Japan; 3 Division of Organ Transplantation, Tohoku University Hospital, Sendai, Miyagi Japan

**Keywords:** Lung transplant, COVID-19, Pneumonia, Vaccine, SARS-CoV-2, Remdesivir, Japan

## Abstract

Lung transplant (LTx) recipients face a significant risk from coronavirus disease 2019 (COVID-19), with elevated hospitalization mortality rates even post-vaccination. While severe acute respiratory syndrome coronavirus 2 (SARS-CoV-2) typically induces pneumonia in even healthy individuals, it can also infect the transplanted lungs of LTx recipients, potentially leading to graft dysfunction. Despite the prevalence of COVID-19 pneumonia in LTx recipients, data on its characteristics and associated risk factors remain limited. This retrospective study analyzed data from LTx recipients at Tohoku University Hospital between January 2001 and November 2023. COVID-19 cases were identified, and patient records, including thoracic computed tomography (CT) evaluations, were reviewed. Patient characteristics, vaccination history, immunosuppressant use, and comorbidities were assessed. Descriptive analysis was utilized for data presentation. Among 172 LTx recipients, 39 (22.7%) contracted COVID-19, with 9 (23%) developing COVID-19 pneumonia. COVID-19 incidence in LTx recipients aligned with national rates, but pneumonia risk was elevated. Delayed antiviral therapy initiation was noted in pneumonia cases. Remdesivir was uniformly administered and remained the primary treatment choice. LTx recipients are susceptible to COVID-19 pneumonia, warranting vigilance and tailored management strategies. Pre-transplant vaccination and prompt COVID-19 diagnosis and treatment are imperative for optimizing outcomes in this population.

## Background

Coronavirus disease 2019 (COVID-19) presents a substantial threat to solid organ transplant (SOTx) recipients, particularly lung transplant (LTx) recipients, who exhibit a notably higher hospitalization mortality rate, even two doses of the SARS-CoV-2 vaccine [1]. Specifically, LTx recipients face an elevated risk of mortality compared to recipients of non-lung SOTx [2]. While COVID-19, caused by the severe acute respiratory syndrome coronavirus 2 (SARS-CoV-2), can induce pneumonia even in otherwise healthy individuals, the emergence of mRNA vaccines has shown promise in reducing the disease's severity [3, 4]. However, LTx recipients have demonstrated a limited antibody response even after receiving two or three doses of the vaccine against SARS-CoV-2 [5, 6]. Furthermore, COVID-19 can precipitate graft infection in LTx recipients, potentially resulting in loss of graft function [7, 8]. Nevertheless, there remains a paucity of data regarding the rates of complications and associated risk factors specifically related to COVID-19 pneumonia in LTx recipients. Therefore, the primary objective of this study is to elucidate the characteristics of LTx recipients who develop complicated pneumonia in the context of COVID-19.

## Method

Patients who underwent LTx at Tohoku University Hospital between January 2001 and November 2023 were included in the study. Medical records of LTRs were retrospectively reviewed up to the end of November 2023. LTRs who tested positive for COVID-19 were identified, and all underwent thoracic computed tomography (CT) evaluation, regardless of the development of pneumonia. Although the specific strain of SARS-CoV-2 was not genetically identified, it was inferred to be the predominant strain circulating in Japan during the respective infection periods, based on data from Japan's National Institute of Infectious Diseases [9]. The diagnosis of COVID-19 relied on the results of reverse transcription-polymerase chain reaction (RT-PCR) testing [5, 10, 11].

For the purpose of this study, "relapse" was defined as the recurrence of symptoms within 90 days after initial improvement [12]. Conversely, cases in which COVID-19 was diagnosed again after a symptom-free interval exceeding 90 days were categorized as "recurrence." Patient characteristics at the time of only the initial infection were collected for LTx recipients who experienced COVID-19 recurrence. Vaccination history, use of immunosuppressants, and the presence of comorbidities such as diabetes mellitus (DM), chronic kidney disease (CKD) [13], and chronic lung allograft dysfunction (CLAD) [14, 15] were evaluated based on the most recent status preceding the onset of COVID-19. Descriptive statistics were used to present variables, including medians with interquartile ranges [IQR] and actual numbers with percentages, as appropriate.

## Results

**Fig. 1:**
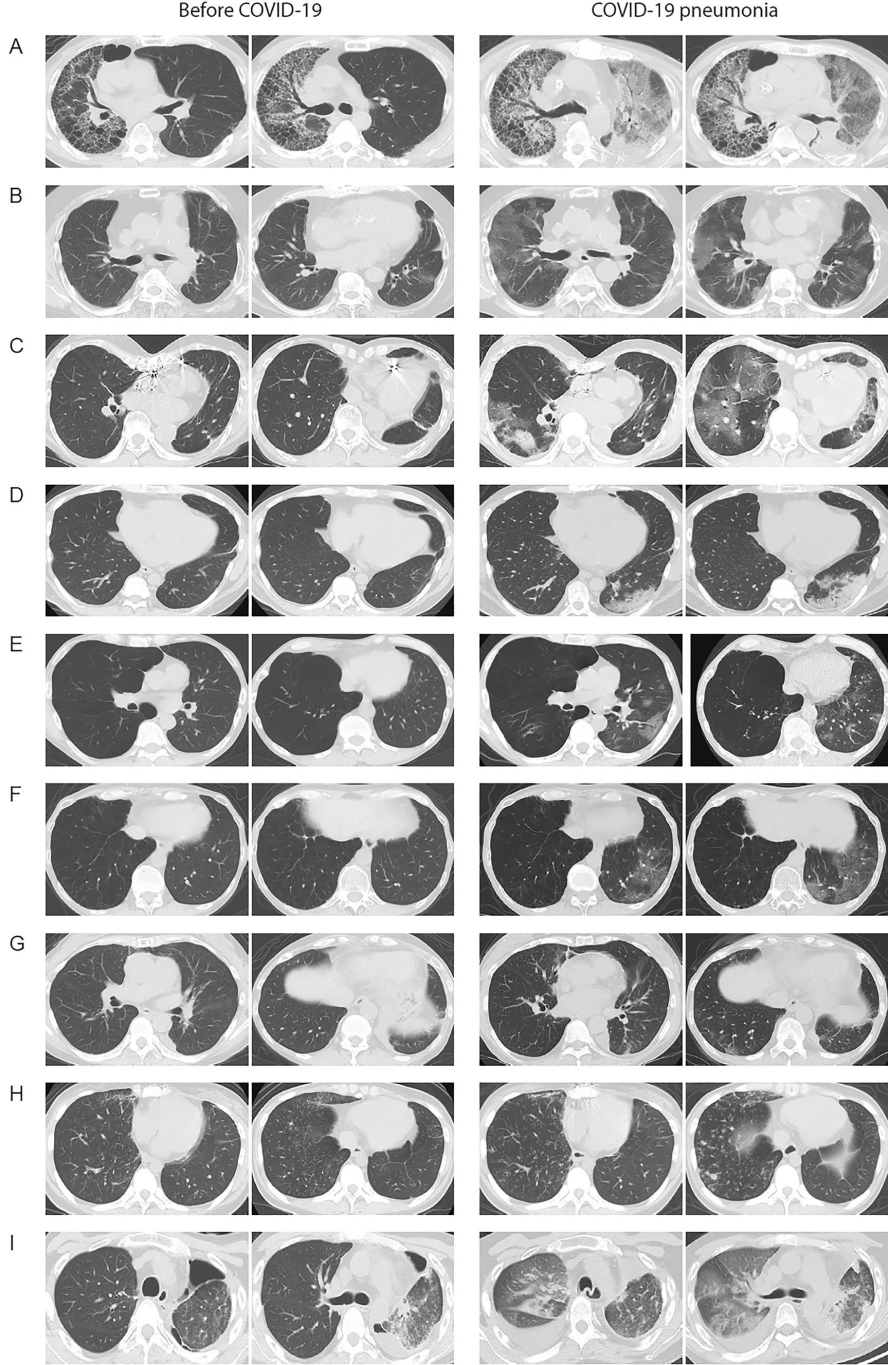
Chest CT findings of lung transplant recipients before and after COVID-19 pneumonia. Representative images include: **A** a male in his 50s, 3 years post left single LTx for idiopathic pulmonary fibrosis; **B** a male in his 50s, 11 years post bilateral LTx for Eisenmenger syndrome; **C** a female in her 60s, 8 years post bilateral LTx for pulmonary alveolar proteinosis; **D** a male in his 30s, 3 years post bilateral LTx for Eisenmenger syndrome; **E** a male in his 50s, 4 years post left single LTx for chronic obstructive pulmonary disease; **F** a female in her 50s, 5 years post left single LTx for lymphangioleiomyomatosis (LAM); **G** a female in her 50s, 9 years post bilateral LTx for LAM; **H** a female in her 20s, 4 years post living-donor lobar LTx for pulmonary arterial hypertension; and **I** a female in her 50s, 12 years post right single LTx. COVID-19: Coronavirus disease 2019 and LTx: lung transplant;

A total of 172 patients who underwent LTx at TUH were included in the study. Among them, 39 patients were diagnosed with COVID-19, resulting in an accumulated incidence of COVID-19 among LTx recipients in Japan of 22.7% (39/172) as of November 2023. Of the 39 patients, 9 (23%) developed COVID-19 pneumonia (CP +), while 30 did not exhibit pneumonia (CP-), as summarized in Table 1. The representative CT images on CP + are shown in Fig. 1. The clinical course of the nine LTx recipients with CP + is summarized as follows:

**Table 1:** Characteristics of lung transplant recipients infected with SARS-CoV-2

Characteristic	COVID-19 + *n* = 39	COVID-19 pneumonia + *n* = 9	COVID-19 pneumonia – *n* = 30
Age at infected, years (IQR)	48 (39–58)	53 (50–57)	45 (39–58)
Sex male, *n* (%)	14 (35.9)	4 (44.4)	10 (33.3)
BMI, kg/m^2^ (IQR)	17.5 (15.6–21.2)	17.8 (17.2–23.5)	17.1 (15.3–21.1)
LTx type, *n* (%)			
Single	17 (43.5)	4 (44.4)	13 (43.3)
Bilateral	16 (41.0)	4 (44.4)	12 (40.0)
Living-donor	6 (15.4)	1 (11.1)	5 (16.6)
Underlying disorder, *n* (%)			
Obstructive	13 (33.3)	4 (44.4)	9 (30.0)
Fibrosis	8 (20.5)	2 (22.2)	6 (20.0)
Vascular	11 (28.2)	3 (33.3)	8 (26.7)
Suppurative	4 (10.3)	0	4 (13.3)
Allogenic	3 (7.7)	0	3 (10.0)
Vaccinated prior to LTx, *n* (%)	4 (10.3)	0	4 (13.3)
Vaccinated after LTx ¶, *n* (%)	30 (76.9)	8 (88.8)	22 (73.3)
T/C prior to COVID-19, *n* (%)	9 (23.1)	4 (44.4)	5 (16.7)
TAC trough level, ng/mL (IQR)	8.4 (6.4–9.9)	8.2 (6.4–9.0)	8.8 (6.1–10.3)
MMF AUC level, µghr/mL (IQR)	31.5 (27.0–43.4)	38.4 (34.1–56.7)	29 (25.2–42.2)
Diabetes mellitus, *n* (%)	4 (10.3)	3 (33.3)	1 (3.3)
Chronic kidney disease, *n* (%)	2 (5.1)	0	2 (6.7)
Chronic lung allograft dysfunction, *n* (%)	10 (25.6)	2 (22.2)	8 (26.7)

*COVID-19* Coronavirus disease 2019 *BMI* body mass index *IQR* interquartile range *LTx* lung transplant *T/C* tixagevimab/cilgavimab *TAC* tacrolimus *MMF* mycophenolate mofetil, and *AUC* area under the curve recipients who were vaccinated at least twice after LTx before developing COVID-19

*Case 1* A 53-year-old male underwent left single LTx for idiopathic pulmonary fibrosis 3 years ago (Figure 1-A). He had received four doses of an mRNA vaccine and one set of tixagevimab/cilgavimab (T/C). On the 6th day after fever onset, he was diagnosed with COVID-19 and initially received symptomatic treatment. By the 10th day, he developed progressive respiratory failure; a chest CT scan revealed extensive lung infiltrates on the graft. Despite intubation, extracorporeal circulation, and treatment with remdesivir, antibacterial agents, and steroid pulse therapy, his hypoxemia did not improve. He passed away on the 27th hospital day.

*Case 2* A 58-year-old male underwent bilateral LTx for Eisenmenger syndrome 11 years ago (Figure 1-B). He had received three doses of the mRNA vaccine and one dose of T/C. One day after symptom onset, he tested positive for SARS-CoV-2 and was treated with remdesivir for 5 days at our center. Two weeks after discharge, he experienced a relapse of fever; a CT scan revealed diffuse ground-glass opacities in all lung lobes. Mycophenolate mofetil (MMF) was discontinued, and he received additional remdesivir for 10 days and dexamethasone. He was discharged on the 15th hospital day.

*Case 3* A 63-year-old female underwent bilateral LTx for pulmonary alveolar proteinosis 8 years ago (Figure 1-C). She had received four doses of an mRNA vaccine but not T/C. She developed a fever and tested positive for SARS-CoV-2 on the same day. With no initial drop in lung function, she was treated with molnupiravir, leading to defervescence. However, on the 9th day, she experienced a relapse with a drop in lung function; chest CT revealed pneumonia, and she was hospitalized to receive remdesivir for 5 days. On the 13th day, she continued to experience loss of appetite and weight loss. MMF was discontinued, and she was re-administered remdesivir for 5 days along with dexamethasone. She was discharged 2 months after hospitalization.

*Case 4* A 36-year-old male underwent bilateral LTx for Eisenmenger syndrome 3 years ago (Figure 1-D). He had received two doses of an mRNA vaccine but not T/C. After his family was diagnosed with COVID-19, he developed a fever 2 days later. Despite testing positive for SARS-CoV-2, his lung function remained stable, and he was initially managed with antipyretics, resulting in clinical improvement. On the 21st day, he visited the hospital due to feeling unwell. A CT scan revealed consolidation in the left lower lobe, and he was hospitalized and treated with sotrovimab and remdesivir. He improved and was discharged after 6 days of hospitalization.

Case* 5* A 53-year-old male underwent left single LTx for chronic obstructive pulmonary disease 4 years ago (Figure 1-E). He had received two doses of an mRNA vaccine but not T/C. He developed a fever and tested positive for SARS-CoV-2. Unable to receive antiviral treatment at that time, he followed symptomatic treatment. Fourteen days after symptom onset, he visited the hospital because of shortness of breath; a chest CT scan revealed pneumonia on the graft. He was treated with remdesivir and improved swiftly.

*Case 6* A 53-year-old female underwent left single LTx for lymphangioleiomyomatosis (LAM) 5 years ago (Figure 1-F). She had received four doses of an mRNA vaccine but not T/C. After contracting COVID-19, she was treated with molnupiravir but did not improve. Six days after symptom onset, a CT scan revealed graft pneumonia, requiring treatment with remdesivir upon admission. Despite the worsening of chronic kidney disease, she was discharged 2 months after hospitalization.

*Case 7* A 50-year-old female underwent bilateral LTx for LAM 9 years ago (Figure 1-G). She had received six doses of an mRNA vaccine but not T/C. She developed a fever, tested positive for SARS-CoV-2, and was prescribed molnupiravir. By the 5th day, her symptoms had not improved. Although her lung function was stable, a CT scan revealed pneumonia, leading to hospitalization on the same day. She was treated with remdesivir for 10 days, leading to swift improvement. On the 18th hospital day, her symptoms relapsed. She was then treated with ensitrelvir and discharged on the 24th hospital day.

Case* 8* A 23-year-old female underwent living-donor LTx for pulmonary arterial hypertension 4 years ago (Figure 1-H). As a post-transplant complication, she developed chronic graft infection with *Stenotrophomonas maltophilia* and had several hospitalizations due to exacerbations. She had not received an mRNA vaccine or T/C. She was diagnosed with COVID-19, and a CT scan revealed bronchitis and pneumonia. It was unclear whether this was an exacerbation of pre-existing bronchitis or a new finding due to COVID-19, so she was treated with ceftazidime and remdesivir, to which S. maltophilia was sensitive. Following treatment, her fever subsided and lung function returned to baseline.

Case* 9* A 57-year-old female underwent right single LTx 12 years ago (Figure 1-I). She had received four doses of an mRNA vaccine and one set of T/C. Ten days after developing a fever, she was diagnosed with COVID-19. A chest CT scan revealed pneumonia on the graft, and she was treated with remdesivir and dexamethasone. During her hospitalization, she developed acute kidney dysfunction, leading to severe uremia. Despite various treatments, she passed away 4 months after contracting COVID-19.

Overall, the median age of LTx recipients who developed COVID-19 pneumonia was 53 years (interquartile range [IQR] 50–57), which was slightly higher compared to those without pneumonia, with a median age of 45 years (IQR 39–58). No significant differences were observed in sex distribution, type of lung transplant, or underlying disorders between the CP + and CP- groups. Notably, none of the patients in the CP + group had received an mRNA vaccine prior to transplantation. Following transplantation, both groups received mRNA vaccination, with no discernible difference in immunization rates (88.8% vs. 73.3%).

Regarding the blood concentrations of immunosuppressants, the area under the curve (AUC0-12) for MMF in the CP + group showed a trend toward being higher (median AUC 38.4 µg·hr/mL vs. AUC 29.0 µg·hr/mL) compared to the CP- group. However, there were no significant differences observed in the trough concentration of tacrolimus (median trough 8.2 ng/mL vs. 8.8 ng/mL) between the two groups. While there was a tendency toward a higher proportion of comorbid DM in the CP + group (33.3% vs. 3.3%), no significant differences were noted in the prevalence of CKD (0% vs. 6.7%) or CLAD (22.2% vs. 26.8%) between the two groups, respectively.

The treatment experience is summarized in Table 2. Overall, the median time since LTx to COVID-19 diagnosis was 8 years (IQR 4–11). There was a delay in initiating antiviral therapy in the CP + group, with a median duration of 6 days from the date of infection, compared to within 1 day in the CP- group. Only one patient was infected during the period when the Delta variant was predominant in Japan, while all other cases occurred during the period marked by the dominance of the Omicron variant. All nine patients with COVID-19 pneumonia received hospital treatment, including administration of remdesivir and antibiotics. Among LTx recipients diagnosed with COVID-19, a total of four patients succumbed to the illness, with 2 (22.2%) in the CP + group and 2 (6.7%) in the CP- group. Additionally, relapse was observed in 5 patients (55.5%), all of whom were in the CP + group. Conversely, a decline in forced expiratory volume in one second (FEV1) at 30 days showed no significant difference between the groups.

**Table 2:** Treatments and outcomes of COVID-19 pneumonia

Characteristic	COVID-19 *n* = 39	COVID-19 pneumonia + *n* = 9	COVID-19 pneumonia – *n* = 30
Time since LTx to COVID-19, years (IQR)	8 (4–11)	5 (4–9)	9 (4–12)
Dominant strain of SARS-CoV-2, n (%)			
Wuhan: Jan 2020–Dec 2020	0	0	0
Alfa: Jan 2021–Jul 2021	0	0	0
Delta: Aug 2021–Dec 2021	1 (2.6)	0	1 (3.3)
Omicron: Jan 2022–Nov 2023	38 (97.4)	9 (100)	29 (96.7)
Days since symptom to treatment, days (IQR)	1 (0–2)	6 (0.50–12)	0 (0–1)
Hospital admission, n (%)	26 (66.7)	9 (100)	17 (56.7)
Ventilation, *n* (%)	2 (5.1)	1 (11.1)	1 (3.3)
Use of extracorporeal circulation, *n* (%)	1 (2.6)	1 (11.1)	0
Antibody agents, *n* (%)			
Sotrovimab	2 (5.1)	1 (11.1)	1 (3.3)
Casirivimab/imdevimab	0	0	0
Antiviral agents, *n* (%)			
Molnupiravir	11 (28.2)	3 (33.3)	8 (26.7)
Nirmatrelvir/Ritonavir	0	0	0
Ensitrelvir	1 (2.6)	1 (11.1)	0
Remdesivir	22 (56.4)	9 (100)	13 (43.3)
High-dose prednisolone, *n* (%)	5 (12.8)	4 (44.4)	1 (3.3)
Antibacterial agents, n (%)	10 (25.6)	9 (100)	1 (3.3)
Reduction or discontinuation of MMF, *n* (%)	5 (12.8)	4 (44.4)	1 (3.3)
Outcomes			
Death, *n* (%)	4 (10.3)	2 (22.2)	2 (6.7)
Drop in FEV1 in 30 days, *n* (%)	9 (23.1)	3 (33.3)	6 (20.0)
Relapse, *n* (%)	5 (12.8)	5 (55.5)	0

COVID-19 coronavirus disease 2019 *IQR* interquartile range *MMF* mycophenolate mofetil *FEV1* forced expiratory volume in one second

## Discussion

The study aimed to evaluate the characteristics of LTx recipients with complicated pneumonia in COVID-19. Among the 172 participants, 39 individuals (approximately 23%) were infected with COVID-19. This rate aligns closely with the national infection rate reported by the government of Japan as of May 2023, which was approximately 27% [16]. Therefore, the incidence of COVID-19 in LTx recipients does not appear to significantly differ from that in the general population. However, among the 39 participants who contracted COVID-19, 9 individuals (23%) developed pneumonia. The previous reports have suggested that 46% of LTx recipients infected with SARS-CoV-2 exhibit pneumonia on CT scans [17]. In contrast, the probability of developing pneumonia in the general population, as reported from Japan, is less than 1% [18]. These findings indicate a heightened risk of pneumonia complications on LTx recipients. Although the rate of severe cases of COVID-19 has decreased recently, often allowing for symptomatic treatment only, several reports have highlighted an increased risk of severe outcomes in patients with SOTx or those who are immunosuppressed [19, 20]. In particular, in LTx recipients, pneumonia can directly affect their graft, potentially leading to graft dysfunction. Therefore, it is essential to recognize that SARS-CoV-2 continues to pose a significant threat to LTx recipients, necessitating continued vigilance and appropriate management strategies.

In this study, it was noted that none of the recipients with CP + had received a vaccine prior to transplantation. Vaccination has been widely demonstrated to be effective in preventing COVID-19 and reducing its severity [3, 4]. Assessment of immunity against SARS-CoV-2 often involves evaluating antibody titers, rather than cellular immunity [21]. However, due to lifelong immunosuppression, SOTx recipients frequently exhibit a diminished immune response even after receiving two doses of the vaccine [5, 22, 23], leading to lower antibody titers and an increased risk of severe illness [5, 24]. Our previous study indicated that even after receiving three doses of mRNA vaccines following lung transplantation, only approximately 50% of patients achieved adequate antibody titers [6]. As a result, recent recommendations from the American Society of Transplantation and the International Society for Heart and Lung Transplantation advocate for completing vaccination against SARS-CoV-2 prior to transplantation [25]. Additionally, a recent report suggested that fourth and fifth booster vaccinations could further enhance antibody levels [26]. Therefore, it is currently advisable to ensure completion of vaccination prior to transplantation, and consideration should be given to administering additional doses post-transplantation to optimize immunity against SARS-CoV-2.

Regrettably, there was a trend toward delayed initiation of antiviral agents in patients with CP + , with treatment initiation occurring after a median duration of 6 days from the date of infection, compared to within 1 day in individuals with CP-. International guidelines for COVID-19 management underscore the importance of promptly administering antiviral agents or neutralizing antibodies following symptom onset [27]. According to the National Institutes of Health (NIH) guidelines, molnupiravir and nirmatrelvir/ritonavir should be administered as early as possible within 5 days of symptom onset, while remdesivir initiation is recommended within 7 days [28]. Studies have also suggested that initiating antiviral therapy within 3 days of symptom onset can effectively limit viral replication [29]. Furthermore, delays in diagnosis and treatment initiation have been associated with poorer outcomes, including increased mortality and a heightened risk of severe disease [30, 31]. Recent research has indicated that among LTx recipients who were vaccinated against SARS-CoV-2, those who received early intervention experienced mild symptoms without a clinically significant decline in lung function [32]. At our center, delays in hospital visits following the onset of fever, as well as the initial absence of pneumonia in CP + patients who were treated only symptomatically, likely influenced the treatment delay. Therefore, it is essential for LTx recipients to understand that COVID-19 remains a critical disease requiring immediate medical attention. Additionally, antiviral treatment should be considered as early as possible for patients diagnosed with COVID-19 in this population. Based on these observations, we have implemented a protocol whereby all LTx recipients are requested to purchase a commercial SARS-CoV-2 antigen test kit. If they develop symptoms such as fever, they are instructed to test themselves at home and seek immediate medical attention. In the future, it will be debated whether these measures were ineffective or meaningful, but at present, we believe that early intervention is a key strategy to prevent pneumonia. While it is plausible that delayed treatment initiation contributed to the development of COVID-19 pneumonia in LTx recipients, it remains challenging to definitively ascertain whether early intervention could unequivocally save lives in these patients. Nonetheless, continued vigilance and prompt testing for SARS-CoV-2 infection upon symptom onset are warranted in LTx recipients to facilitate timely intervention and optimize outcomes.

In this study, antiviral agents, including remdesivir, were uniformly administered to all patients. Molnupiravir, however, has been reported not to reduce the frequency of hospitalization or death in vaccinated populations at high risk for COVID-19, and consequently, its usage has been rare at TUH since 2023 [33]. Nirmatrelvir/ritonavir and ensitrelvir, due to their CYP3A4 inhibitory effects, have the potential to increase blood levels of immunosuppressive drugs such as tacrolimus or sirolimus, as well as other prophylactic medications such as voriconazole or itraconazole, which are commonly prescribed in LTx recipients, thereby complicating medication management [34–37]. Conversely, remdesivir does not necessitate consideration of interactions with immunosuppressive drugs and requires the dose-adjustment based on kidney function. Moreover, remdesivir has demonstrated a reduction in the risk of hospitalization or mortality by 87% in patients at risk of severe complications [38], along with decreased mortality rates among hospitalized immunocompromised patients [39]. Therefore, among these agents, remdesivir remains the primary drug to be considered for initial treatment of COVID-19 in LTx recipients.

The small sample size complicates statistical analysis. First, the variability of data significantly impacts the outcomes, increasing the likelihood of results being incidental. This may lead to issues with reproducibility and complicate comparisons with other studies. Furthermore, LTx recipients represent a small group with specific conditions and characteristics, which may limit the applicability of the obtained results to other populations. This situation can diminish the external validity and generalizability of the outcomes. In our study, out of the 39 LTx recipients infected with SARS-CoV-2, 9 (23%) developed COVID-19 pneumonia (CP +), while 30 did not exhibit pneumonia (CP-). This is particularly problematic for the smaller CP + group, where potential biases may occur. Consequently, we were unable to perform statistical analysis to compare CP + and CP-. A recent study with larger sample sizes could potentially address this limitation [40]. Sindu D et al. conducted a single-center analysis involving 195 LTx recipients with COVID-19. In their study, 58.3% of the patients required hospitalization, and 24.1% died, although detailed information on pneumonia complications was not provided. Their analysis was conducted across different stages of the pandemic (analogous to our dominant strain classification), and the larger sample size allowed them to use Cox proportional hazards models for multivariate analysis. They identified risk factors for hospitalization (age over 65 and renal impairment) and mortality (Delta variant and age over 65). This demonstrates that a larger sample size enables more comprehensive analyses. Even though their study is retrospective at a single center, conducting statistical analyses can add clinical significance and make the findings more impactful. This highlights the importance of increasing sample size to enhance the robustness and generalizability of the results.

While our study provides insights into COVID-19 pneumonia on LTx recipients, it is crucial to acknowledge several limitations. Firstly, this research relies on a retrospective analysis from a single center, which may introduce selection bias and limit the generalizability of our findings. Additionally, the small sample size may affect the reliability of our results as documented above. Furthermore, the comparison between patients with CP + and those with CP- was hindered by the limited number of COVID-19 pneumonia cases among LTx recipients (only nine), rendering a meaningful comparative assessment unfeasible. Moreover, the specific strain of SARS-CoV-2 was not genetically detected in our study. It is essential to recognize that various factors, including individual patient characteristics and advancements in medical treatments, can influence the management and outcomes of COVID-19 complications in LTx recipients. To address these limitations, multicenter prospective studies should be considered to gather more robust outcomes data. However, establishing such studies poses challenges, as each center may have different protocols for managing LTx recipients with COVID-19. Additionally, the dominant strains of SARS-CoV-2 change over time, and the characteristics of LTx recipients are heterogeneous.

In summary, LTx recipients exhibit a similar infection rate of SARS-CoV-2 compared to the general population but are more prone to developing COVID-19 pneumonia affecting the graft. Vaccination is recommended for LTx recipients both before and after transplantations. Consequently, early diagnosis and prompt treatment of COVID-19 may contribute to better outcomes, but further studies are warranted.

## Data Availability

The datasets used and/or analyzed during the current study are available from the corresponding author on reasonable request.

## Abbreviations

COVID-19: Coronavirus disease 2019
SARS-CoV-2: Severe acute respiratory syndrome coronavirus 2
IQR: Interquartile range
LTx: Lung transplant
CYP3A4: Cytochrome P450 3A4
